# Monovalent vaccination with inactivated SARS-CoV-2 BA.5 protects hamsters against Omicron but not non-Omicron variants

**DOI:** 10.1038/s41541-023-00776-x

**Published:** 2023-11-20

**Authors:** Chon Phin Ong, Kaiming Tang, Pak-Hin Hinson Cheung, Hongzhuo Zhang, Tze-Tung Tang, Yaqian Xue, Junjue Wang, Kelvin Kai-Wang To, Shuofeng Yuan, Zi-Wei Ye, Dong-Yan Jin

**Affiliations:** 1 School of Biomedical Sciences, Li Ka Shing Faculty of Medicine, The University of Hong Kong, Pokfulam, Hong Kong, China; 2 State Key Laboratory of Emerging Infectious Diseases, Carol Yu Centre for Infection, Department of Microbiology, School of Clinical Medicine, Li Ka Shing Faculty of Medicine, The University of Hong Kong, Pokfulam, Hong Kong, China; 3 Centre for Virology, Vaccinology and Therapeutics, Hong Kong Science and Technology Park, Shatin, Hong Kong, China; 4 Department of Infectious Disease and Microbiology, The University of Hong Kong-Shenzhen Hospital, Shenzhen, China

**Keywords:** Viral infection, SARS-CoV-2, Inactivated vaccines

## Abstract

We compared the protective effects of inactivated SARS-CoV-2 vaccines derived from the ancestral and the currently circulating BA.5.2 strains against infection with multiple variants in Syrian golden hamsters. Vaccination with BA.5.2 effectively protected against infection with the Omicron subvariants including XBB.1, but not the Alpha or Delta variant. In contrast, hamsters vaccinated with the ancestral strain demonstrated decent neutralization activity against both the Omicron and non-Omicron variants. Our findings might instruct future design and formulation of SARS-CoV-2 vaccines.

Vaccination against SARS-CoV-2 has been very effective in the protection against severe disease and death, but it was less successful in the prevention of viral transmission^1,2^. To deal with the emergence of SARS-CoV-2 variants of concern (VOCs) that exhibit increased immune evasiveness, viral strains used for vaccine production need to be updated periodically^3^. Whereas BA.2 and BA.5 subvariants of SARS-CoV-2 Omicron were consecutively predominant worldwide in early and mid-2022, they gave way to other subvariants such as BQ.1, BQ.1.1, XBB.1, XBB.1.5 and BF.7 in some parts of the world, but BA.5 and BA.2 continue to dominate in other areas^2,4^. One future direction is to formulate one single type of vaccine for annual booster immunization against the circulating strains, coupled to influenza vaccination^3^. However, questions remain unanswered as to whether a monovalent or bivalent SARS-CoV-2 vaccine should be used and whether a monovalent vaccine against the predominant strain could have effective protection against sufficiently broad range of VOCs. To shed light on these questions, in this study we asked whether vaccination of Syrian golden hamsters with inactivated SARS-CoV-2 Omicron BA.5.2 subvariant might provide good protection against emerging subvariants, such as XBB.1, and VOCs that circulated before the arrival of the Omicron variant. Side-by-side comparison with the inactivated vaccine derived from the ancestral strain was made.

SARS-CoV-2 variants propagated in VeroE6-TMPRSS2 cells for 5 days were inactivated. As verified by immunoblotting, the purified virions of the ancestral (Wuhan-1) and the Omicron BA.5.2 strains of SARS-CoV-2 contained abundant viral spike (S) and N proteins (Fig. 1a, Supplementary Fig. 1). Syrian golden hamsters (6 per group) were vaccinated twice with 3 μg each of inactivated virions of BA.5.2 or ancestral strains in a prime-boost scheme with an interval of 14 days (Fig. 1b). Anti-N antibodies were equally robust in animals vaccinated with BA.5.2 and ancestral strains after 28, 77, and 101 days (Fig. 1c). In stark contrast, the levels of anti-RBD of the ancestral strain were considerably lower in BA.5.2-vaccinated hamsters than in animals vaccinated with the ancestral strain at all three time points. Consistently, sera from BA.5.2-vaccinated hamsters displayed much less pronounced inhibitory activity on the binding of ACE2 to the RBD of the ancestral strain in the surrogate virus neutralization test (sVNT) (Fig. 1d). Furthermore, when live virus microneutralization assay (LVMNA) was carried out with multiple SARS-CoV-2 VOCs, sera of BA.5.2-vaccinated hamsters exhibited strong neutralizing activity against BA.5.2 as well as other Omicron subvariants BA.1, BA.2.12.1, BQ.1.1 and XBB.1 at 28 days post-vaccination (Fig. 1e, Supplementary Fig. 2). The neutralizing activity against BA.1 decreased to right above basal after 77 days, when the neutralization of BA.5.2, BA.2.12.1, BQ.1.1 and XBB.1 remained robust. However, these sera had almost lost the ability to neutralize Alpha or Beta strain at the same time point, with only 2 out of 6 sera capable of neutralizing. Opposite to this pattern, sera from animals vaccinated with the ancestral strain effectively neutralized the Alpha and Delta variants at both early and late time points. Moreover, their neutralizing activity against BA.1, BA2.12.1 and BA.5 was well above the basal level. However, the sera could marginally neutralize XBB.1 (Fig. 1e). Interestingly, the protective efficacy differences between ancestral- and BA.5.2-vaccinated groups become increasingly prominent as time progresses.

**Fig. 1:**
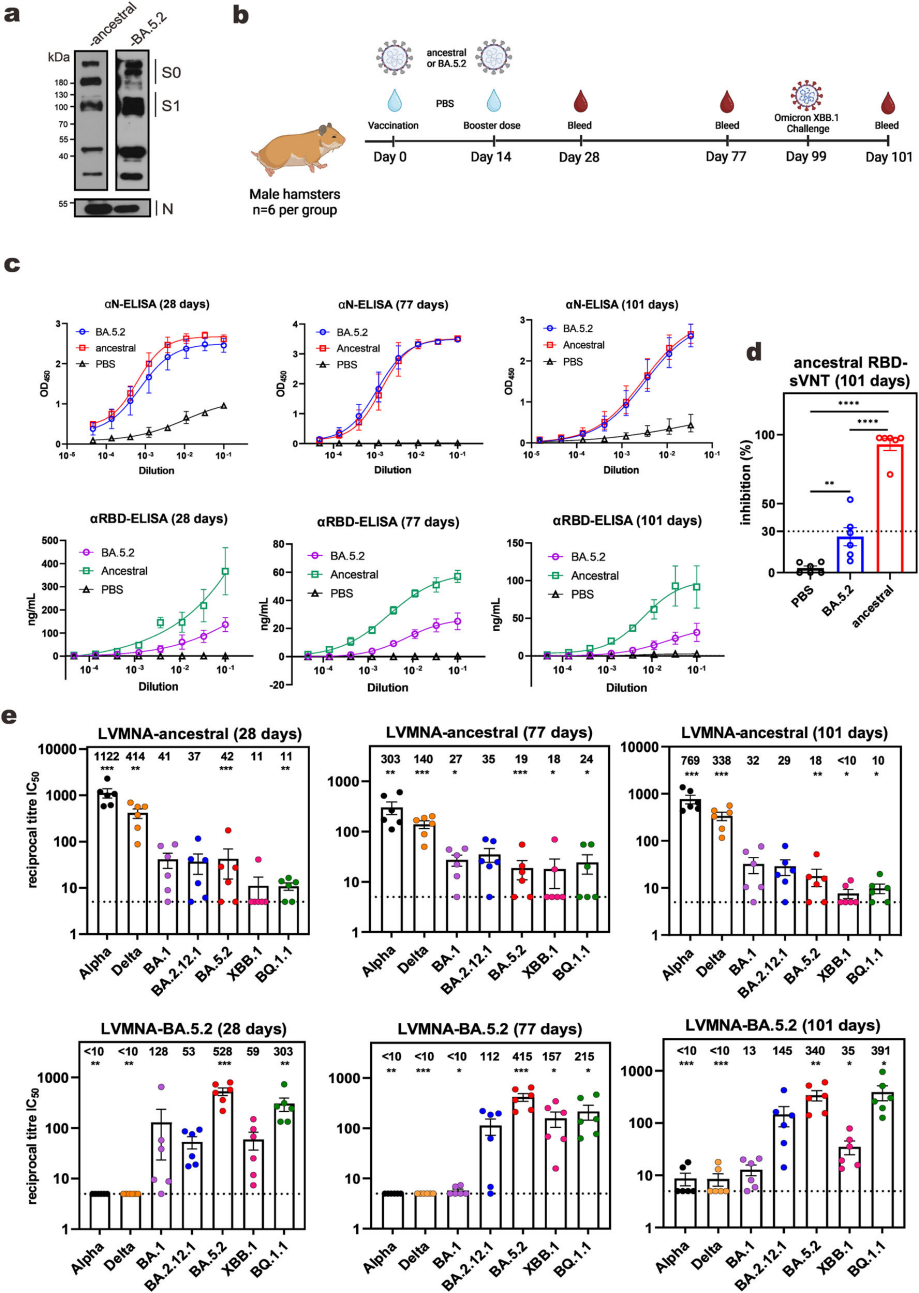
Neutralizing activity in vaccinated hamsters. **a** Immunoblot for proteins in SARS-CoV-2 virions purified by sucrose-gradient centrifugation for vaccine preparation. The uncleaved S0 and the cleaved S1 are indicated. All virus stocks have been verified by Nanopore sequencing after passaging in VeroE6-TMPRSS2 cells. Rabbit monoclonal anti-RBD is from ThermoFisher (HL257). Mouse monoclonal anti-N was home-made. **b** Vaccination scheme of hamsters. Schematic diagram was created using BioRender.com. **c** ELISA assays. Hamster sera were collected at 28, 77, and 101 days post-vaccination for analysis of anti-RBD (αRBD) or anti-N (αN). For anti-RBD, a standard curve plotted from known concentrations of an anti-SARS-CoV-2 RBD monoclonal antibody (HL257, MA5-26353, ThermoFisher Scientific) was used to determine antibody concentration. Results are from three independent experiments. Error bars represent mean ± standard deviation (SD). **d** sVNT assays. Error bars represent mean ± standard error mean (SEM) (*n* = 6). **e** LVMNA assays. Limit of detection (LOD) is 5 (dotted line). Error bars represent mean ± SEM (*n* = 6). *: *P* < 0.05. **: *P* < 0.01. ***: *P* < 0.001. ****: *P* < 0.0001. IC_50_: 50% inhibitory concentration.

When challenged with XBB.1, significantly lower numbers of SARS-CoV-2 sub-genomic envelop (E) gene copies were found in both lung and tracheal tissues of vaccinated hamsters (Fig. 2a). However, there was no significant inter-group difference in nasal wash samples. It remained to be clarified whether residual and dead RNA might persist in the nasal cavity. Thus, we went on to perform plaque assays in parellel to quantify infectious virions in the samples. As expected, amounts of infectious virions in lung, tracheal and nasal wash samples of the BA.5.2-vaccinated animals, as measured by plaque assays, were remarkably lower (Fig. 2b). This reduction was less pronounced in lung and trachea of animals vaccinated with the ancestral strain. In addition, mRNA expression levels of proinflammatory cytokines interleukin-4 (IL-4) and IL-6 as well as chemokine CCL-17 increased in the lung of BA.5.2-vaccinated hamsters (Fig. 2c). Histopathological analysis revealed minimal immune cell infiltration and alveolar wall edema in the lung of these animals. Histological scoring of the lung sections reveals significant severity in the PBS-treated hamsters, but not vaccinated hamsters (Fig. 2d).

**Fig. 2:**
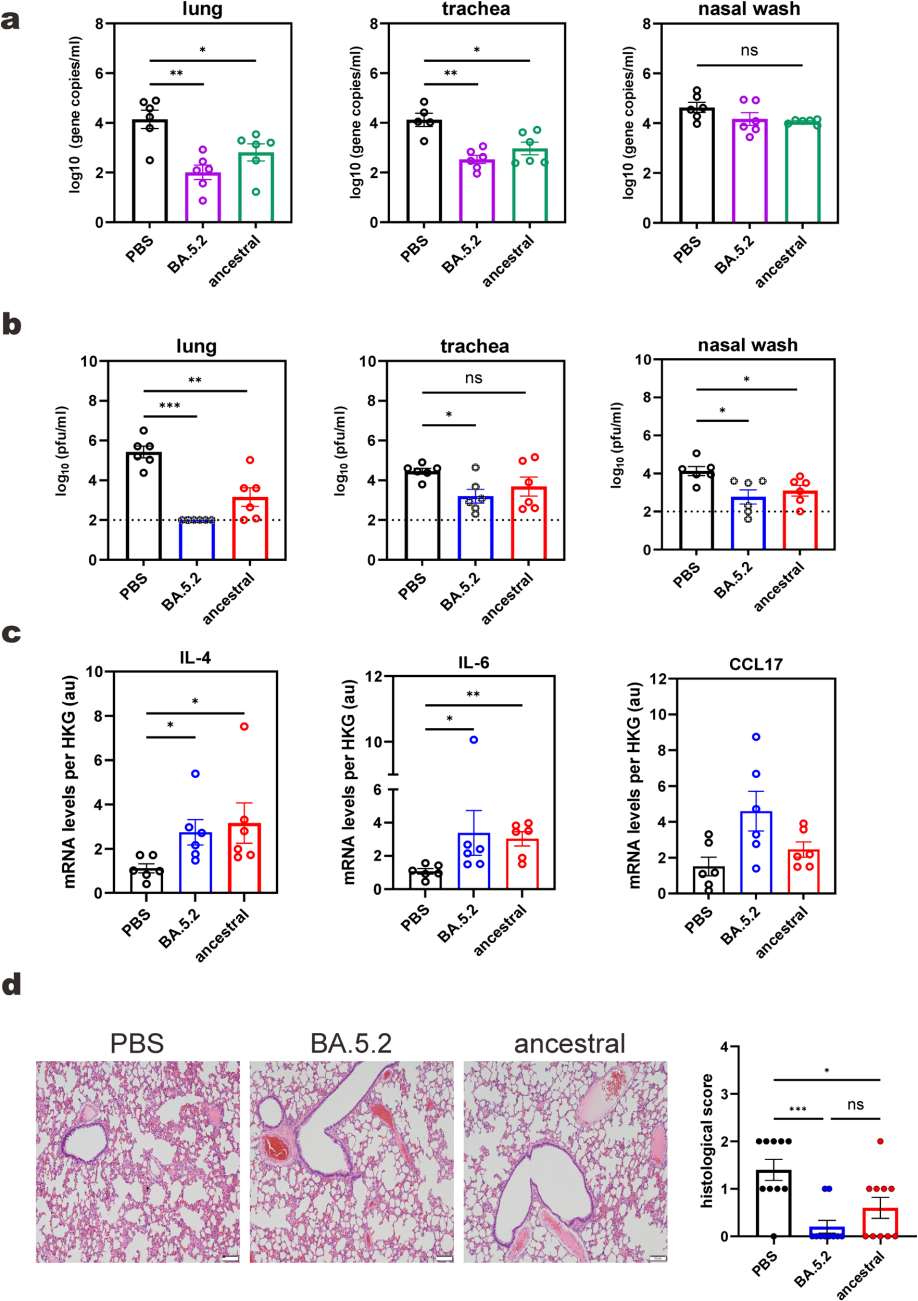
Viral replication in vaccinated and challenged hamsters. **a** Subgenomic E gene copies in respiratory samples. Hamsters were vaccinated and challenged with XBB.1 at 99 days post-vaccination. Samples were collected for RT-qPCR analysis at 2 dpi. One-way ANOVA was performed to determine significant differences between groups. Error bars represent mean ± SEM (*n* = 6). **b** Viral titers in respiratory samples at 2 dpi by plaque assays. The limit of detection (LOD) is 100. One-way ANOVA was performed to determine significant difference between groups. Error bars represent mean ± SEM. **c** Cytokine and chemokine profiling of the lung at 2 dpi by RT-qPCR. One-way ANOVA was performed to determine significant difference between groups. Error bars represent mean ± SEM (*n* = 6). **d** Hematoxylin and eosin staining of the lung at 2 dpi. Histological scores were derived as described^15^. Scale bar: 100 µm. One-way ANOVA was performed to determine significant difference between groups. Error bars represent mean ± SEM (*n* = 8). *: *P* < 0.05. **: *P* < 0.01. ***: *P* < 0.001. *ns* not significant (*P* > 0.05). pfu plaque forming unit. au arbitrary unit. HKG house-keeping gene (β-actin).

Whether a monovalent or bivalent vaccine should be used for post-pandemic vaccination against SARS-CoV-2 remains to be understood^5^. Bivalent mRNA vaccines expressing S of both ancestral and BA.5 strains induce broadly neutralizing antibodies against existing VOCs in animals and humans^6,7^, providing good protection against symptomatic infection with BA.5 and XBB.1 subvariants. Our demonstration of the cross-protection of Omicron subvariants by vaccination with inactivated BA.5 is generally consistent with this notion. Plausibly, vaccination with BA.5 should provide long-lasting protection against other Omicron subvariants, including emerging subvariants and subvariants to be emerged. It will be of interest to see whether the bivalent vaccine could be replaced by one single monovalent vaccine directed against the predominant strain currently circulating in the world.

Our finding that vaccination with inactivated BA.5 was less effective in protecting against non-Omicron variants suggests that inclusion of a non-Omicron strain in the SARS-CoV-2 vaccine might be desirable^5,8^. Most people in the general population were infected by the Omicron variant in 2022, but much less might have been exposed to the non-Omicron variants^9^. Particularly, some children and a small subset of adults might still be both unvaccinated and uninfected. Whereas the Delta variant has been found to be highly fit in immunologically naïve population^10^, the Omicron variant might have outcompeted the Delta variant only in vaccinated and/or infected people^11^. In other words, pre-existing immunity against non-Omicron variants in humans could have driven the evolution of the Omicron, which is not only most diverged from the non-Omicron variants but also most immune evasive^1,2^. It will be intriguing to investigate whether the absence of a non-Omicron variant in the SARS-CoV-2 vaccine could facilitate the re-selection and re-emergence of the Delta variant, which is more pathogenic than Omicron.

Exclusion from the composition of the updated SARS-CoV-2 vaccine of the ancestral strain that is no longer circulating has been proposed^3^. Before we can reach a conclusion on this, several important questions must be answered. First, whether previously circulated strains of SARS-CoV-2 could re-emerge and re-circulate as those of influenza viruses remains to be seen. Second, whether strong pre-existing immunity against non-Omicron variants acquired through vaccination and/or natural infection might guard against the re-emergence of the non-Omicron variants as suggested above or emergence of another Delta-like variant should be clarified. Finally, whether the pre-existing immunity against non-Omicron variants would be long-lasting and even enhanced via booster vaccination and/or natural infection with an Omicron subvariant merits further investigations^12^. Nevertheless, inclusion of a non-Omicron strain in the SARS-CoV-2 vaccine might not be unwise.

## Methods

### Viruses

SARS-CoV-2 Omicron XBB.1 variant was isolated from respiratory tract specimens of laboratory-confirmed COVID-19 patient in Hong Kong. Other clinical isolates of SARS-CoV-2 have been described previously^13,14^. VeroE6-TMPRSS2 cells were cultured until confluency. Viruses were inoculated the next day and incubated at 37 °C for 5 days. Viral supernatant was harvested and inactivated with paraformaldehyde to a final concentration of 0.2% at 4 °C for 5 more days. Virions were concentrated and purified as described^5^. Purified inactivated SARS-CoV-2 containing 3 μg of protein was mixed at 1:1 ratio with Imject^TM^ Alum Adjuvant (Thermo Scientific, 77161) before injected intramuscularly into Syrian golden hamsters. A booster dose was given after 14 days.

ELISA assays for SARS-CoV-2 spike receptor binding domain (RBD) or nucleocapsid, surrogate virus neutralization test (sVNT) using cPass^TM^ SARS-CoV-2 Neutralization Antibody Detection Kit, and live virus microneutralization assay (LVMNA) were carried out as previously described^15^.

### Infection of Syrian golden hamsters

All protocols for animal experiments have been approved by the Committee on the Use of Live Animals in Teaching and Research (CULATR) of the University of Hong Kong (Reference code: CULATR 5754-21). They were also performed in accordance with the standard operating procedures of Biosafety Level 3 animal facilities. Male Syrian golden hamsters aged 4-6 weeks old were used. At day 99 post-vaccination, hamsters anesthetized with ketamine (200 mg/kg) and xylazine (10 mg/kg) intraperitoneally were intranasally challenged with 10^5^ PFU (in 50 μL) of SARS-CoV-2 Omicron XBB.1 subvariant. At 2 days post-infection (dpi), hamsters were sacrificed via intraperitoneal injection of dorminal (200 μL) for virological and histopathological evaluation. RT-qPCR and Western blotting were performed as described^5,15^. Primers used are listed in Supplementary Table 1.

### Statistical analysis

Statistical significance was assessed by unpaired two-tailed Student’s *t* test or one-way analysis of variance (ANOVA) with Welch’s correction and Dunnett’s post-hoc test using Prism version 8.4.3.

### Reporting summary

Further information on research design is available in the Nature Research Reporting Summary linked to this article.

### Supplementary information

Supplementary information

REPORTING SUMMARY

## Data Availability

Data supporting the findings of this study are available in this paper, in the Supplementary Information, or from the corresponding author upon reasonable request.
