# Supplementary material for: Monovalent vaccination with inactivated SARS-CoV-2 BA.5 protects hamsters against Omicron but not non-Omicron variants
Source: NPJ Vaccines. 2023 Nov 20;8:177. doi: 10.1038/s41541-023-00776-x (PMC10662430; doi:10.1038/s41541-023-00776-x)
Supplement: Supplementary file 1 — Supplementary information [file 41541_2023_776_MOESM1_ESM.pdf]

Supplementary Information

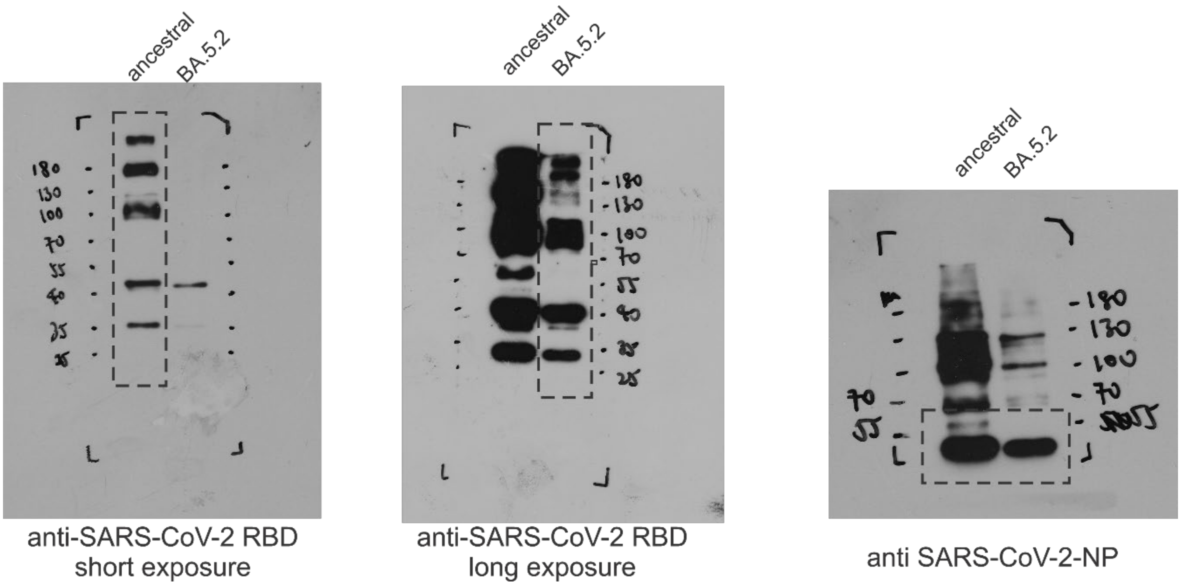

Supplementary Fig. 1 Uncropped/unprocessed Western blot image. Rabbit monoclonal anti-SARS-CoV-2 RBD (ThermoFisher) and in-house mouse monoclonal anti-SARS-CoV-2 NP were used in immunoblotting of purified virions.

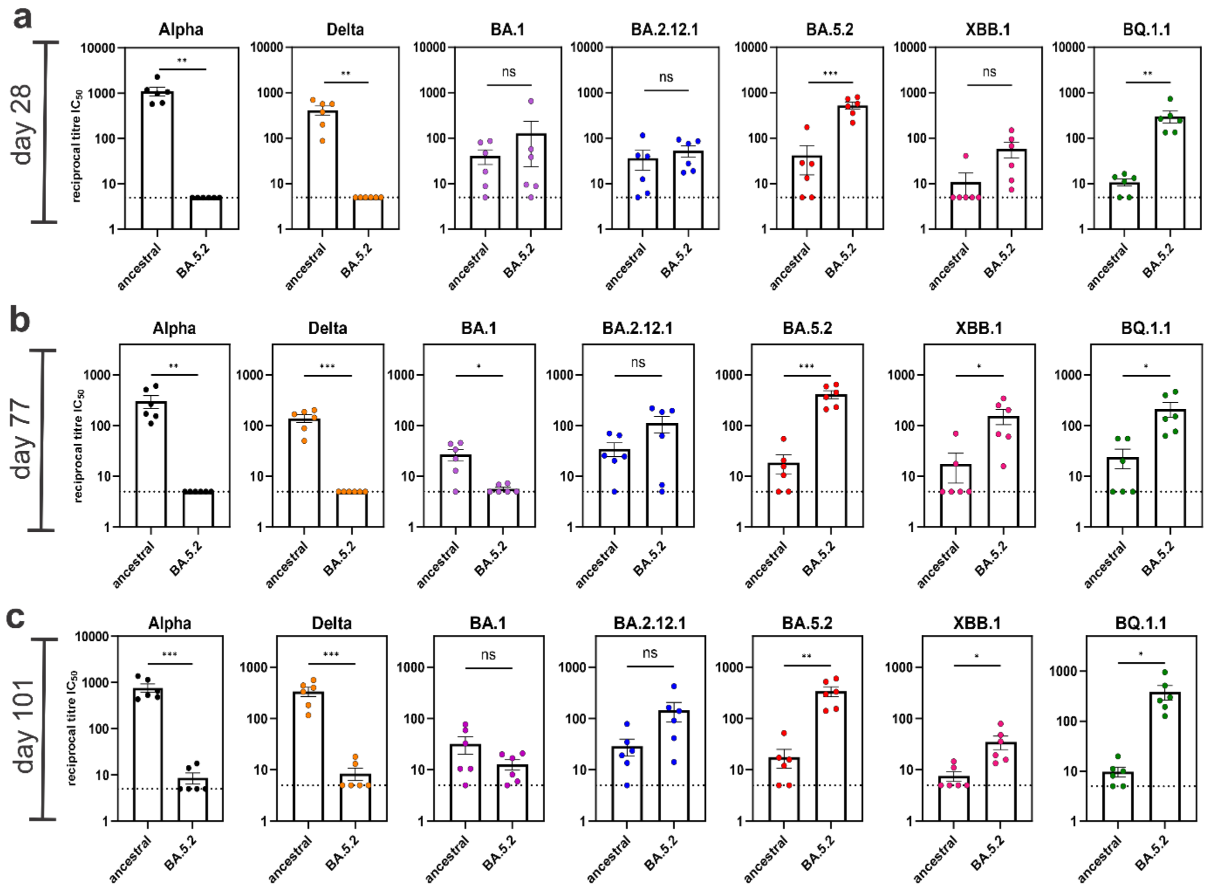

Supplementary Fig. 2 Statistical analysis of LVMNA results. Unpaired two-tailed Student's t-test was performed with LVMNA against SARS-CoV-2 Alpha, Beta, BA.1, BA.2.12.1, BA.5.2, XBB.1 and BQ.1.1 at days 28 (**a**), 77 (**b**), and 101 (**c**) post-vaccination. Error bars represent mean ± SEM (n = 6). ∗: $P < 0.05$. ∗∗: $P < 0.01$. ∗∗∗: $P < 0.001$. *ns*: not significant ($P > 0.05$).

Supplementary Table 1 Primer list.

**SARS-CoV-2**	
SARS-CoV-2 Subgenomic E (sgE) probe	/5HEX/AC ACT AGC C/ZEN/A TCC TTA CTG CGC TTC G/3IABkFQ/
SARS-CoV-2 sgE Forward	CGATCTCTTGTAGATCTGTTCTC
SARS-CoV-2 sgE Reverse	ATATTGCAGCAGTACGCACAC
**Hamster**	
IL-4 Forward	ACAGAAAAAGGGACACCATGCA
IL-6 Reverse	GAAGCCCTGCAGATGAGGTCT
IL-6 Forward	CCT GAA AGC ACT TGA AGA ATT CC
IL-6 Reverse	GGT ATG CTA AGG CAC AGC ACA CT
CCL17 Forward	CGA GTG CTG CCT GGA GAT C
CCL17 Reverse	TGA TGG CCT TCT TCA CAT GC
β-actin Forward	ACTGCCGCATCCTCTTCCT
β-actin Reverse	TCGTTGCCAATGGTGATGAC

Primers used in RT-qPCR for quantification of viral titer and hamster lung cytokines/chemokines are listed (5′ – 3′).
